# Supplementary material for: Prominent signatures of energy transfer in action-detected spectra of a cyanobacterial photosynthetic protein
Source: Chem Sci. 2026 Aug 13;17(39):18771–81. doi: 10.1039/d6sc03582e (PMC13487457; doi:10.1039/d6sc03582e)
Supplement: Supplementary file 1 [file SC-017-D6SC03582E-s001.pdf]

Supplementary Information (SI) for Chemical Science.
This journal is © The Royal Society of Chemistry 2026

Supporting Information

Prominent Signatures of Energy Transfer in Action-Detected Spectra of a Cyanobacterial Photosynthetic Protein

Sayan Ghosh,[†] Amitav Sahu,[‡] Stephanie Gonzalez Migoni,[¶] Thomas L C Jansen,[*,¶] and Vivek Tiwari[*,†]

†*Solid State and Structural Chemistry Unit, Indian Institute of Science, Bangalore, Karnataka 560012, India*

‡*Present Address: Division of Chemical Physics and NanoLund, Lund University, Box 124, 221 00 Lund, Sweden*

¶*Zernike Institute of Advanced Materials, University of Groningen, Nijenborgh 3, 9747 AG Groningen, Netherlands.*

E-mail: t.l.c.jansen@rug.nl; vivektiwari@iisc.ac.in

Section S1: Theoretical Methods

A coarse-grained simulation approach is employed to study the spectral signatures of exciton annihilation in APC. Below we describe this approach briefly. Further details can be found in our recent work.[1] First, we divide the system in segments according to their intermolecular coupling strengths. We then use a stochastic model to build a Frenkel exciton Hamiltonian. This Hamiltonian is later used in response function calculations under the doorway-window framework using the Numerical Integration of the Schrödinger Equation (NISE) approach[2] to determine the 2DES. Additionally, F-2D spectra were obtained using the recently developed coarse-grained 2DES approach in NISE[1,3] and by computing the quantum yields associated with each segment.

The molecular coordinates of the APC structure were obtained from the protein data bank (pdb code 7sc7).[4] The structure is shown in Figure S1. We store the positions for a single trimer consisting of three α and three β phycocyanobilin (PCB) pigment molecules, with a total of 43 atoms constituting each bilin. A Frenkel exciton model is used to define the Hamiltonian that describes the collective excited states of the system as:[5]

$$H(t) = \sum_{n} \epsilon_n(t) B_n^\dagger B_n + \sum_{n,m\neq n} J_{nm} B_n^\dagger B_m, \tag{S1}$$

where $B_n^\dagger$ and B_n are the Pauli creation and annihilation operators, respectively of molecule n. Moreover, $\epsilon_n(t)$ is the excitation energy of the molecule n. Its time dependence accounts for energy fluctuations due to the dynamic environment.

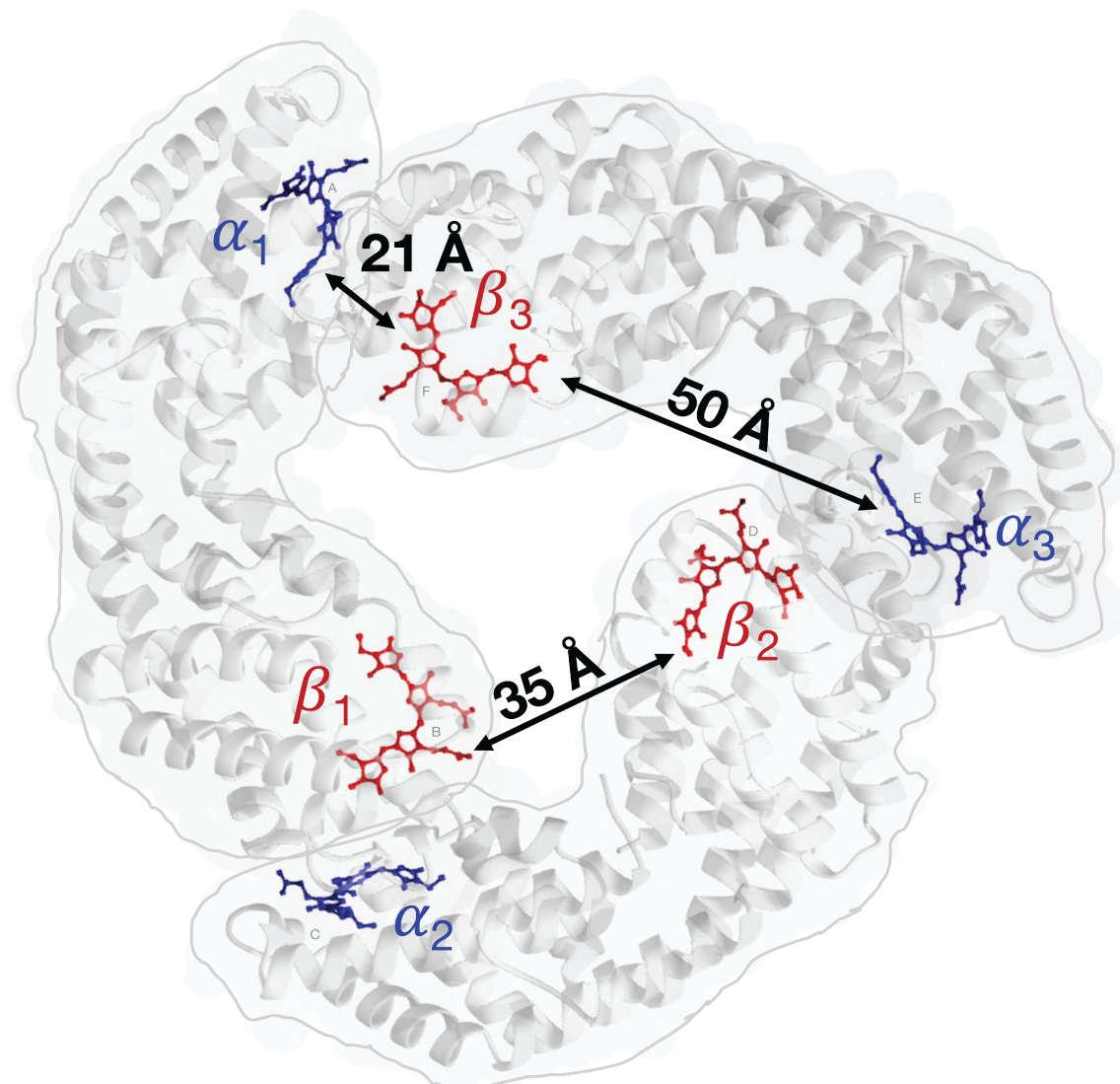

Figure S1: **Crystal structure (PDB ID:7sc7).** APC $(\alpha\beta)_3$, composed of three $\alpha\beta$ monomer separated by thin grey lines. Within an $\alpha\beta$ monomer, chromophores are separated by ~ 50 Å, whereas the distance between α (blue) and β (red) of the nearest monomer is ~ 21 Å.

These energy fluctuations can be generated using various schemes such as molecular dynamics simulations,[6] first principle calculations,[7] or a stochastic model.[8] Here we use the latter, in particular, an overdamped Brownian oscillator model fully characterized by a time-correlation function with the parameters described below. Finally, J_{nm} is the electronic coupling between molecules n and m, which we treat here as a constant for simplicity. As such, we use the point-dipole coupling approximation given by[9]

$$J_{nm} = \frac{1}{4\pi\epsilon_0}\left(\frac{\vec{\mu}_n \cdot \vec{\mu}_m}{r_{nm}^3} - 3\frac{(\vec{\mu}_n \cdot \vec{r}_{nm})(\vec{\mu}_m \cdot \vec{r}_{nm})}{r_{nm}^5}\right), \tag{S2}$$

where $\vec{\mu}_n$ is the transition dipole moment of pigment n, and $\vec{r}_{nm}$ is the vector joining pigments n and m. Alternative coupling schemes may also be easily implemented into the definition of the Hamiltonian.

The time-dependent excitation energies fluctuate around the average site energies energies ϵ_n. Two distinct average site energies were used for these calculations, namely 16060 cm^{-1} for the α-chromophores and 15300 cm^{-1} for the β-chromophores. Furthermore, the

pigments were coupled to an overdamped Brownian oscillator with correlation times of 296 fs for α-chromophores and of 238 fs for β-chromophores. These values were obtained from a spectroscopic model found in literature.[10] Their respective reorganization energies were however adjusted to better fit our experimental absorption spectrum resulting in $\lambda_\alpha = 720$ cm^{-1} and $\lambda_\beta = 141$ cm^{-1}. The couplings J_{nm} in Eq. S2 are computed according to the point-dipole approximation with a transition dipole moment of $|\mu|$=12 Debye.[11]

For defining the response functions, we follow the previously established coarse-grained approach,[1,3] where we introduce the segment dependent quantum yields to describe the dynamics during t_4 and the emission of photons. Consequently, the expression derived for the coarse-grained F-2DES response function is:

$$\begin{aligned} R^{\parallel}_{FD}(t_1,t_2,t_3) = \sum_{\alpha,\beta,\gamma,\delta} \sum_{i,j}^{N_S} & A^{\parallel}_{\alpha\beta\gamma\delta} P_{ji}(t_2)[D^{R}_{i,\alpha\beta}(t_1) + D^{NR}_{i,\alpha\beta}(t_1)] \\ & \{ \sum_{d\in\{GB,SE,EA\}} Q^{(1)}_{j} W^{d}_{j,\delta\gamma}(t_3) + Q^{(2)}_{jj} W^{EA}_{j,\delta\gamma}(t_3) \\ & + \sum_{k\neq j} [2Q^{(1)}_{j} W^{GB}_{k,\delta\gamma}(t_3) - Q^{(2)}_{jk} W^{GB}_{k,\delta\gamma}(t_3)] \} \end{aligned} \tag{S3}$$

Here $Q^{(1)}_j$ represents the quantum yield of a single exciton state starting on segment i. And $Q^{(2)}_{jk}$ represents the quantum yield associated to a bi-exciton state occurring at segments j and k. The sum over $d \in \{GB, SE, ESA\}$ represents the contributions from the rephasing and non-rephasing GB, SE, and ESA1 diagrams. For the ESA2 diagrams, the weight factors are the quantum yields $Q^{(2)}_{jj}$, where jj represents the situation where two excitons are present on the same segment after the application of the last laser pulse. Respectively, $D_i(t_1)$ and $W_j(t_3)$, are the window and doorway functions. Additionally, $P_{ji}(t_2)$ represents the transfer function and $A^{\parallel}_{\alpha\beta\gamma\delta}$ represents the weight factors for parallel polarization.[12]

In the following, we describe the computation of the quantum yields corresponding to each segment in our APC system. Note that these quantum yield estimates are based on parameters which are directly taken from the reported literature without any adjustable

parameter in the APC rate model described in Figures 5a,b. Previous literature reports annihilation lifetime τ_{A1} of 67 ps,[13] a fluorescence lifetime τ_f of 1.87 ns,[14] and a quantum yield $Q^{(1)}$ of 0.7.[15] From these literature values we obtain the radiative k^R and non-radiative k^N rates as well as the annihilation rate k^{A1}, which will be used in our calculations and are connected through the relations

$$Q^{(1)} = \frac{k^R}{k^R + k^N} = \frac{k^R}{k^f}. \tag{S4}$$

Here, assuming all molecules to have the same radiative and non-radiative rates. The quantum yield of each segment will determine its corresponding contribution to the overall signal as described in detail in Ref. 1. In this case, each pigment molecule was regarded as a separate segment, rendering six segments in total. Now, we consider two possible scenarios after the fourth pulse interaction with the sample: either the system will contain a single excitation, or it may contain two.[8] In the first case, once the system has fully relaxed, it can emit with a quantum yield $Q^{(1)}$, which may reach unity when every excited state results in the emission of one photon. In the alternative case, the system can formally emit up to two photons, resulting in a maximum quantum yield $Q^{(2)}$ of 2. Thus, we will have 6 values of $Q^{(1)}$, one per each pigment representing its singly excited manifold; and a total of 21 values of $Q^{(2)}$, one per each possible pair of pigments representing the possible coherence configurations. We can then group these quantum yield factors into 8 categories, for which the values of $Q^{(1)}$ and $Q^{(2)}$ are listed in Table S1.

Table S1: Quantum yields $Q^{(1)}$ and $Q^{(2)}$ obtained as the average of all possible segments or segment combinations. A prime superscript denotes an exciton located on a neighboring site; the absence of a prime indicates that both excitons occupy the same site.

$Q^{(1)}$		$Q^{(2)}$			
α	β	$\alpha\alpha'$	$\beta\beta$'	$\alpha\beta$	$\alpha\beta$'
0.70	0.70	0.8294	0.8293	0.8292	0.8294

Table S2: Rate matrix obtained from the multi-chromophoric Förster resonance energy transfer (MC-FRET) estimation of energy transfer rates. All rates are given in units of (picosecond)$^{-1}$.

	**A**α_1	**D**β_3	**B**β_2	**E**α_3	**C**α_2	**F**β_1
**A**α_1	-1.46E-01	1.14E+06	3.82E+01	2.75E+02	6.54E+02	5.81E+00
**D**β_3	3.32E+04	-4.52E-01	7.09E-01	5.08E-02	9.80E-01	3.10E+00
**B**β_2	1.11E+00	7.09E-01	-4.65E-01	1.33E+02	2.34E-01	1.68E+00
**E**α_3	3.46E+02	2.19E+00	5.75E+03	-4.85E-02	4.76E+02	5.24E+01
**C**α_2	7.69E+02	3.95E+01	9.35E+00	4.42E+02	-1.89E-01	1.19E+04
**F**β_1	1.68E-01	3.09E+00	1.67E+00	1.20E+00	2.95E+02	-9.01E-01

Section S2: Experimental Methods

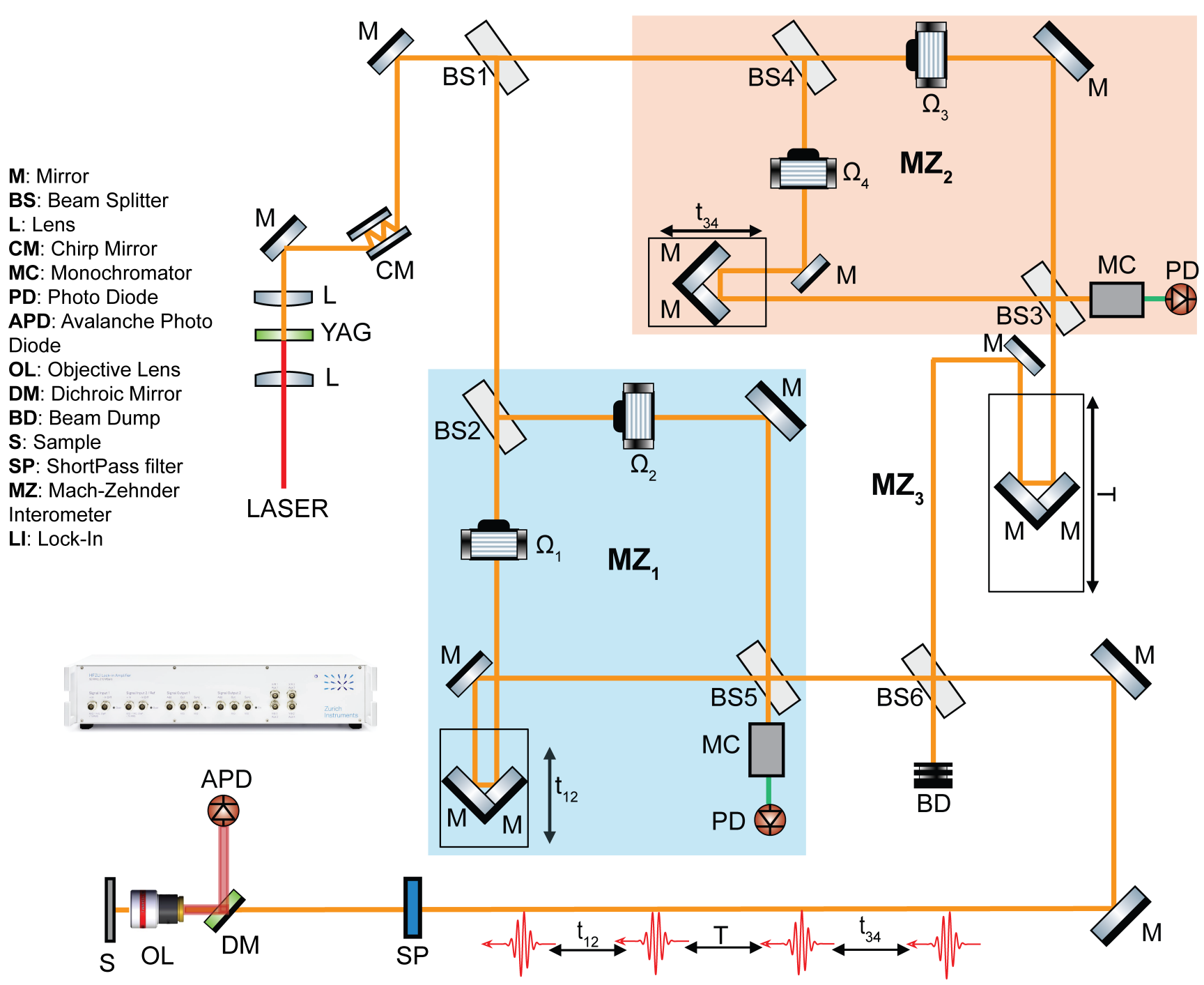

Figure S2: **F-2D and F-PP experimental setup.** The setup implements the acousto-optic phase modulation approach of Marcus and co-workers[16] with a white-light continuum (WLC) input as demonstrated in our previous[17] work. Some graphical elements used in the schematics were adapted from the ComponentLibrary by Alexander Franzen and are used under the Creative Commons Attribution–NonCommercial 3.0 Unported License (CC BY-NC 3.0).

The F-2DES and F-PP setup in Figure S1 implements the acousto-optic phase modulation scheme introduced by Marcus and co-workers,[16] using a white-light continuum (WLC) input. Full experimental details are provided elsewhere.[17] Briefly, laser pulses of energy $\sim$ 1 μJ centered at 1040 nm from a 1 MHz Yb:KGW laser (Spectra Physics Spirit One) are

focused into an 8 mm thick YAG crystal to generate the WLC. The continuum is spectrally separated from the fundamental using an 825 nm short-pass (SP) filter and subsequently routed through chirped mirrors (CMs) for partial pre-compensation of dispersion at the sample. The WLC is then split into pump and probe arms, each containing a Mach–Zehnder interferometer (MZ1 and MZ2). Within each interferometer, the pulses are further divided into phase-modulated pulse pairs. The inter-pulse delays (t_{12} and t_{34}) are controlled via the respective delay stages. Each pulse is modulated at a distinct radio frequency (Ω_1, Ω_2 for the pump; Ω_3, Ω_4 for the probe), such that upon recombination at beam splitters (BS3 and BS5), the pump and probe pulse pairs are modulated at the difference frequencies Ω_{12} and Ω_{34}, respectively. The two pulse pairs are subsequently recombined at BS6, and the pump–probe delay (T) is controlled mechanically.

In the biased sampling scheme,[17] the T stage is translated at a constant velocity for each (t_{12}, t_{34}) delay combination. The resulting four collinear pulses pass through an OD 4, 675 nm SP filter and a 660 nm dichroic mirror (DM), and are focused onto the sample using a 40× reflective objective (LMM-40x-P01, Thorlabs). In both F-2D and F-PP experiments, the sample is continuously circulated through a 500 μm pathlength cuvette using a peristaltic pump at a flow rate of 48 mL/s. The 1/e^2 focal spotsize is $\sim$60 μm. The total pump and probe pulse energy is $\sim$50 pJ in each MZ interferometer. Assuming 600 nm photons, this amounts to a pump fluence of $\sim$5.3 x 10^{16} photons/m^2 compared to the lowest fluence of $\sim$8 x 10^{19} photons/cm^{-2} in the intensity dependent pump-probe measurements of ref.,[13] using which the (67 ps)$^{-1}$ annihilation rate in APC was estimated.

Linear fluorescence signals modulating at Ω_{12} and Ω_{34} are Fourier transformed to yield the Fourier-transform fluorescence excitation (FT-FLEX) spectra for the pump and probe arms, respectively. The nonlinear fluorescence signal, modulating at linear combinations ($\pm\Omega_{12} + \Omega_{34}$), is collected in a confocal geometry using an avalanche photodiode (APD) and

isolated via phase-sensitive lock-in detection. Rephasing and non-rephasing contributions are detected simultaneously using two parallel channels of a lock-in amplifier (Zurich Instruments HF2LI) and summed to obtain the absorptive 2D spectrum. The reconstructed F-2D spectrum, obtained from the direct product of the individual FT-FLEX spectra collected with MZ1 and MZ2, is shown in Figure S4a. Consistent with Figure 1, the maximum of the reconstructed spectrum aligns with the FLEX maximum along the excitation axis and with the intersection of FLEX (blue) and emission (EMS, red) along the detection axis.

For the F-PP measurements, the pump interferometer delay is fixed ($t_{12} = 0$), while the probe delay (t_{34}) is scanned from 0 to 70 fs in 2 fs steps. At each t_{34} position, the pump–probe delay (T) is continuously scanned up to 1 ps at a velocity of 0.03 mm/s. During data processing, the T axis is binned to yield an effective T step size of 5 fs. The $N =$ 36, $t_{12/34}$ points are zero-padded to 128 points prior to Fourier transfrom, with the resulting frequency resolution of $\sim$ 130 cm^{-1} in the F-2D spectra in Figure 1. The true frequency resolution with allowed $2N$ zero-padding is $\sim$ 232 cm^{-1} and is system limited due to rapid dephasing. The extra zero-padding allows us to choose a multiple pixel band to integrate around the upper and lower exciton regions. The F-2D experiment requires $\sim$7 min per 2D spectrum, whereas the complete F-PP measurement takes total $\sim$57 mins for 200 T points.

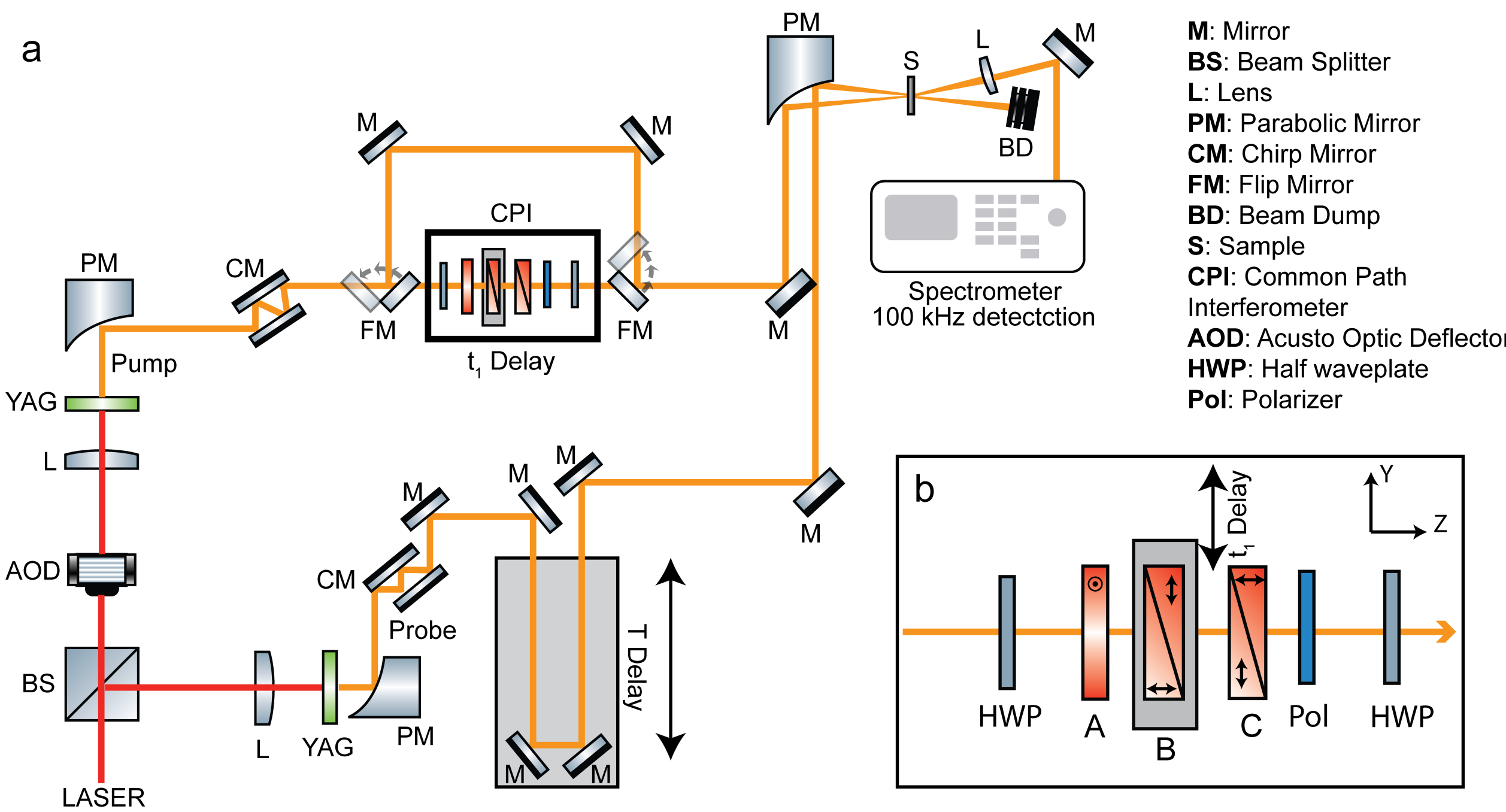

Figure S3: (a) **C-2D and C-PP Experimental Setup.** The setup implements the 100 kHz shot-to-shot scheme described in our previous works.[18,19] Some graphical elements used in the schematics were adapted from the ComponentLibrary by Alexander Franzen and are used under the Creative Commons Attribution–NonCommercial 3.0 Unported License (CC BY-NC 3.0). (b) Common-path interferometer used to generate the pump pulse pair.

Details of the 100 kHz shot-to-shot detection scheme for C-2D and C-PP measurements have been described previously.[18,19] Briefly, separate WLCs are generated for the pump and probe arms. For the probe, ~ 1 μJ pulses at 1040 nm from a 100 kHz Yb:KGW amplifier are focused into an 8 mm thick YAG crystal. For the pump, the fundamental beam is first passed through a crystalline quartz acousto-optic deflector (AOD) to enable 50 kHz modulation, and subsequently focused into a 4 mm thick YAG crystal to generate the WLC. Both pump and probe continua are pre-compensated for dispersion using two pairs of chirped mirrors (CMs).

A birefringent common-path interferometer (CPI) generates the pump pulse pair with a continuously scanned coherence delay (t_1), while the probe pulse is delayed (T) using a computer-controlled mechanical delay stage. The pump and probe beams are focused onto the sample using a 90° off-axis parabolic mirror (PM) with a 10 cm focal length. The sample is contained in a 200 μm pathlength cuvette. To mitigate photodamage, the cuvette is

translated back and forth at a velocity of 1.3 mm/s. The $1/e^2$ focal spotsize is $\sim$ 55 μm. The pump and probe pulse energy is 1 nJ and 0.9 nJ, respectively. The OD before versus after the experiment showed negligible changes (Figure S4d).

After the sample, the transmitted probe is directed to a spectrograph, while the pump is blocked. Shot-to-shot detection is performed using a 100 kHz line scan CCD camera (Teledyne e2v, AViiVA) where the probe shots are recorded at 100 kHz such that the alternate shots correspond to pump-on versus pump-off. In C-2D experiments, the coherence delay t_1 is scanned continuously from -5 fs to 75 fs at a velocity of 0.4 mm/s, yielding a binned time step of ~0.135 fs. The $N = 558$ binned t_1 points are zero-padded to $4N$ points, with the resulting frequency resolution of ~108 cm^{-1} in the C-2D spectra in Figure 3. The true frequency resolution with allowed $2N$ zero-padding is ~216 cm^{-1} and is system limited due to fast dephasing. In C-PP measurements, the pump–probe delay T is scanned from -0.5 to 1.6 ps at a velocity of ~0.076 mm/s, resulting in a binned step size of 5 fs. For C-PP measurements, the pump pulse is not routed through the CPI, and the number of chirped mirror reflections is reduced accordingly. A C-2D experiment requires ~2.5 min per 2D spectrum, whereas the complete C-PP measurement takes total ~3 mins for 432 binned T points. Further details of the electronic setup and data acquisition are provided in refs.[18,19]

Signal Linearity

To rule out higher-order signal contributions,[20] we have also conducted pulse energy dependent measurements of the non-linear signal in our coherent pump-probe (C-PP) data recorded as a function of incident pump pulse energy, while the probe pulse energy is fixed at 0.9 nJ, as in the experiments. As shown in Figure S6, independent linear fits performed at both T points exhibit linear scaling with comparable slopes and intercepts. Details of the fitting are provided in Table S5. The strict linear scaling of the non-linear signal confirms that

our coherent spectroscopy experiments operate within the linear response regime. Crucially, this validation directly extends to our action-detected measurements. In our fluorescence-detected 2D (F-2D) and pump-probe (F-PP) setups, the total pulse energy is limited to approximately 50 pJ per interferometer arm. This is 20x lower than the already low pulse energies used in the coherent experiments (1 nJ and 0.9 nJ for pump and probe, respectively).

Data Analysis

For the F-PP data, the whole dataset was globally fitted using a three-exponential model represented by:

$$F_{\mathrm{PP}}(T,\lambda)=\sum_{i=1}^{3}A_i(\lambda)\exp(-k_iT) \tag{S5}$$

Owing to symmetric signals[21] in F-PP for $T<0$, the initial signal drop in the pulse overlap region was accounted for by including an additional fast exponential decay time constant in the global fitting model. The floated parameters led to 26(0) fs time constant after the global fit, where the parenthesis represents fitting error on the last digit. The timescale for downhill electronic energy transfer from the upper to the lower exciton was extracted at 182(1)fs. A long time constant, determined to be ∼59 ps, was required to account for the slow population decay that were not fully sampled within our ∼1 ps T scan window. The residuals for these fits are shown in Figure S5 and do not show any systematic trends beyond the pulse overlap region. The fitting parameters are summarized in Table S3. In Figure 2b of the main text, a three-pixel region with a width of ∼130 cm^{-1} around 16138 cm^{-1} and 15226 cm^{-1} was integrated and plotted as the upper and lower exciton, respectively. The fit residuals are shown in Figure S5.

The C-PP data was globally fitted using GloTarAn[22,23] with a bi-exponential model convoluted with an instrument response,

$$C_{\mathrm{PP}}(T,\lambda)=\sum_{i=1}^{2}\left[\exp(-k_iT)\otimes\mathrm{IRF}(\mu,\sigma_{\mathrm{FWHM}})\right]A_i(\lambda) \tag{S6}$$

where i is the index of the exponential decay component, $A_i(\lambda)$ represents the decay-associated spectrum (DAS) for the ith time constant, and $\otimes\mathrm{IRF}(\mu,\sigma_{\mathrm{FWHM}})$ indicates convolution with an assumed Gaussian instrument response function defined by its center position (μ) and full width at half maximum (σ_{FWHM}). Note that chirp correction was done as a separate step before the global fit such that μ is not a function of λ. Although the experimental

data was collected up to a delay stage window of 1 ps, a slow time constant of 28 ps was necessary to achieve a good fit. A comparison between the C-PP spectrum at $T = 0.5$ ps and the 224 ± 3 fs DAS is presented in Figure S4c. The DAS corresponding to the 224 fs component exhibits a clear dispersive lineshape indicating downhill energy transfer. Details of the fitting parameters are provided in Tables S3 and S4. The residuals for upper and lower exciton decays in Figure 4b are shown in Figure S5.

Table S3: Global Fitting Parameters for F-PP data in Figure 2. The parenthesis shows the rounded off error.

**Parameters**	**Time Constant**
τ_1	26 (0) fs
τ_2	182 (1) fs
τ_3	59 (13) ps

Table S4: Global Fitting Parameters for C-PP data in Figure 4. The parenthesis shows the rounded off error.

**Parameters**	**Time Constant**
IRF Position (μ)	-5 (0) fs
IRF Width (σ_{FWHM})	23 (0) fs
τ_1	224 (3) fs
τ_2	28 (1) ps

Figure S4b compares the C-PP and F-PP spectra at $T = 200$ fs. Both spectra are normalized at the lower exciton. The upper-to-lower exciton amplitude ratio for C-PP and F-PP is 0.171 and 0.063, respectively. The 2.7× larger lower exciton amplitude and presence of ∼10% negative ESA signal near DP_U in Figure 3 partly accounts for the approximately 3× larger amplitude growth on the lower exciton for the case of C-PP in Figure 4 of the main text.

Table S5: Linear regression parameters for pulse energy dependence of the nonlinear signal

**Delay** (T)	**Slope** (m)	**Intercept** (c)
0.3 ps	0.1825 ± 0.0049	0.0335 ± 0.0055
2.0 ps	0.1792 ± 0.0101	0.0324 ± 0.0112

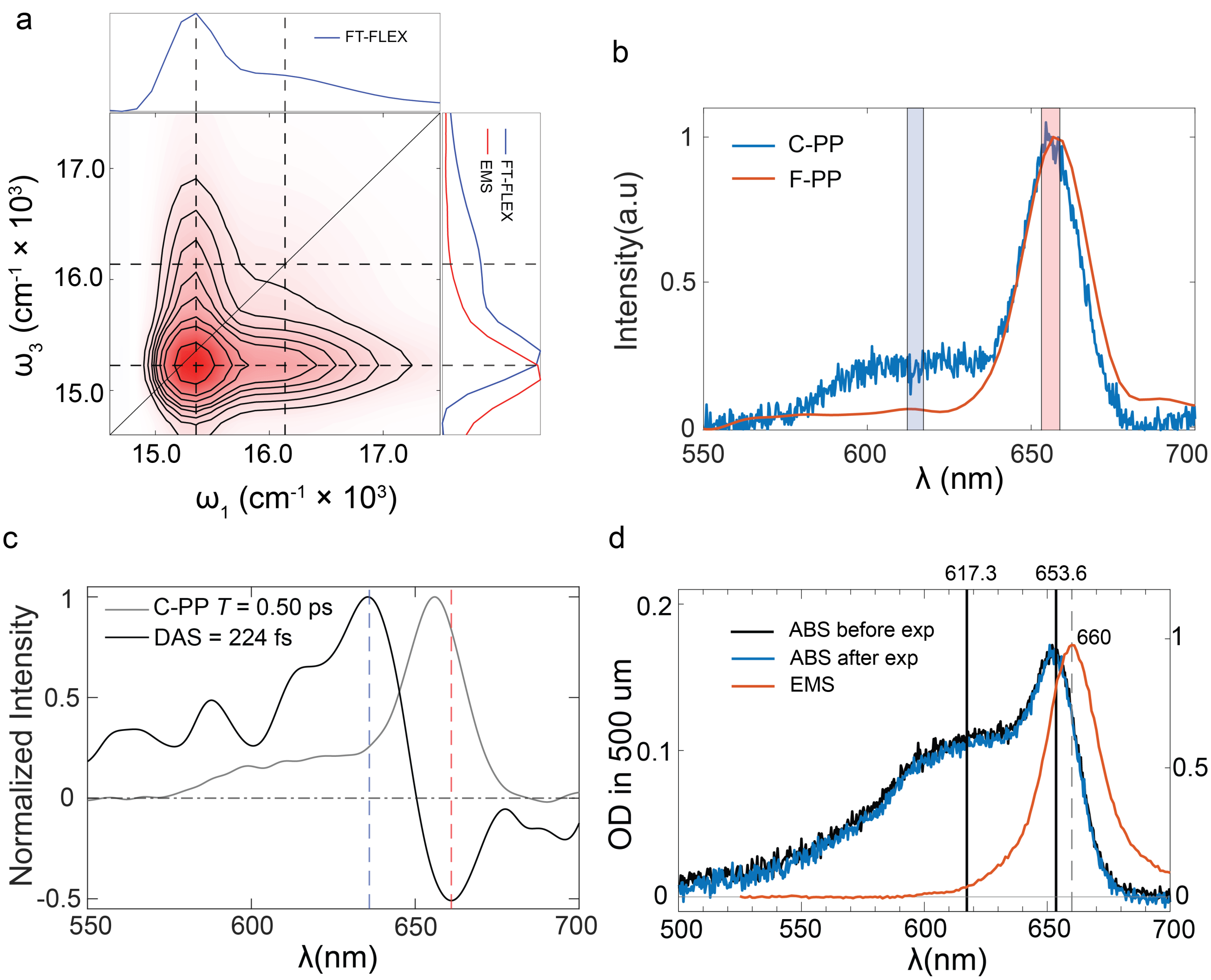

Figure S4: (a) Reconstructed F-2D spectra using a direct product of the Fourier transform fluorescence-excitation (FT-FLEX) spectra measured in the pump MZ interferometer, with the addition of EMS and FT-FLEX measured in the probe MZ interferometer. The maximum of the reconstructed 2D spectrum passes through the maximum of FLEX (blue) along the excitation frequency and the intersection of FLEX (blue) and EMS (red) along the detection frequency. The upper exciton is ∼3× weaker than the lower exciton in the FT-FLEX spectra, which leads to a ∼9× weaker upper exciton in the reconstructed 2D. (b) Comparison of the C-PP and F-PP spectra at $T = 200$ fs. Both spectra are normalized at the lower exciton. The two exciton bands are shaded as blue and red bands. (c) Decay associated spectrum (DAS) of 224±3 fs time constant is overlaid with the C-PP spectrum at $T = 0.50$ ps. (d) A representative absorption spectrum measured before and after the experiments is shown. The sample corresponds to the cross-linked APC.[24] The emission spectrum is also overlaid and peaks at 660 nm expected[24] from the $(\alpha\beta)_3$ trimer in native APC. The solid black lines in the absorption correspond to the upper and lower exciton peak positions determined from prior time-resolved spectroscopy studies[25,26] on native APC.

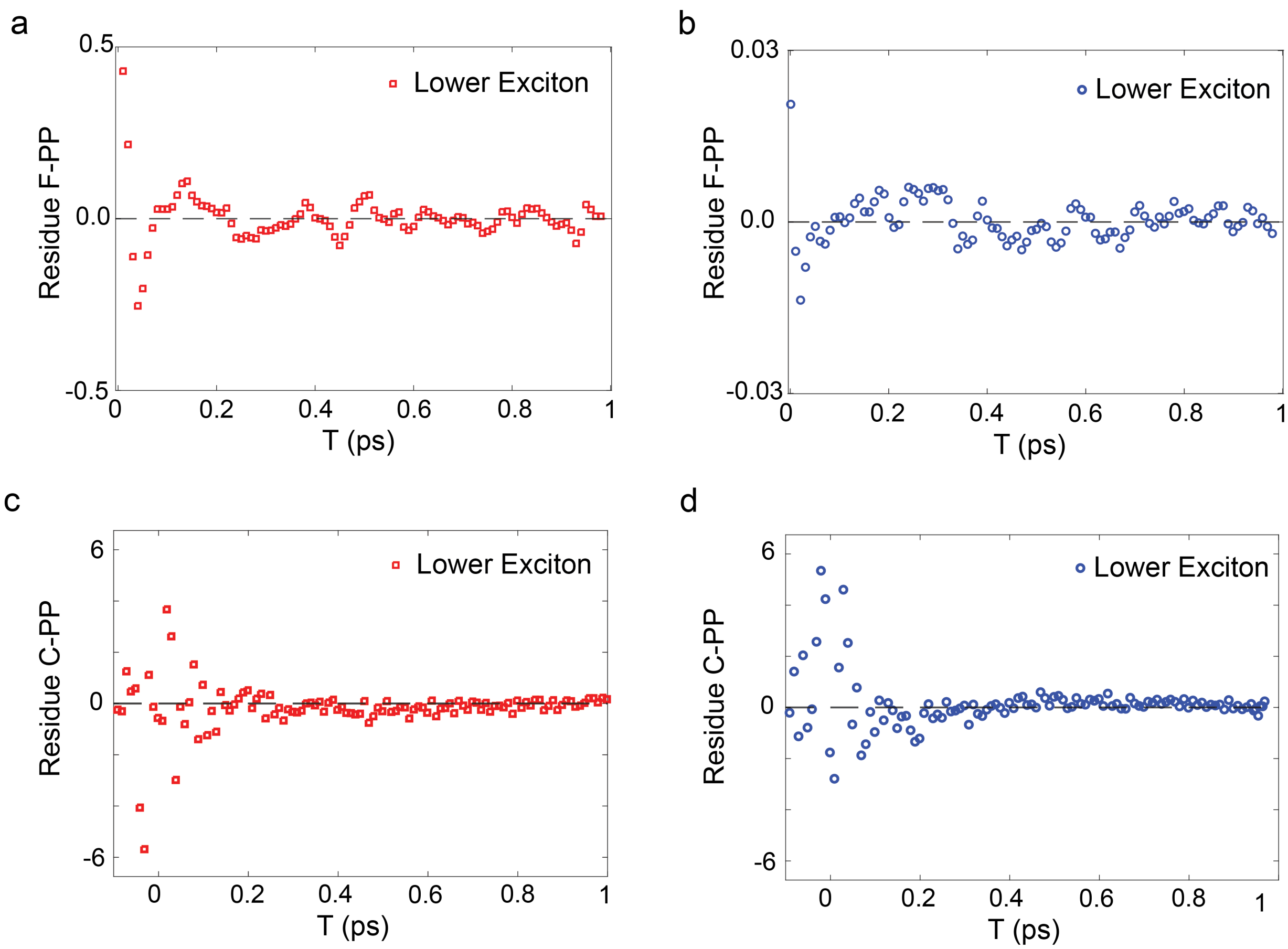

Figure S5: (a, b) Residual plots for the fitting shown in Figure 2b in the main text for the F-PP data. The scale is kept same as Figure 2b. (c, d) Residual plots for the fitting shown in Figure 4b in the main text for the C-PP data. The scale is kept same as Figure 4b. Beyond the pulse overlap region, the residuals do not show any large systematic trends.

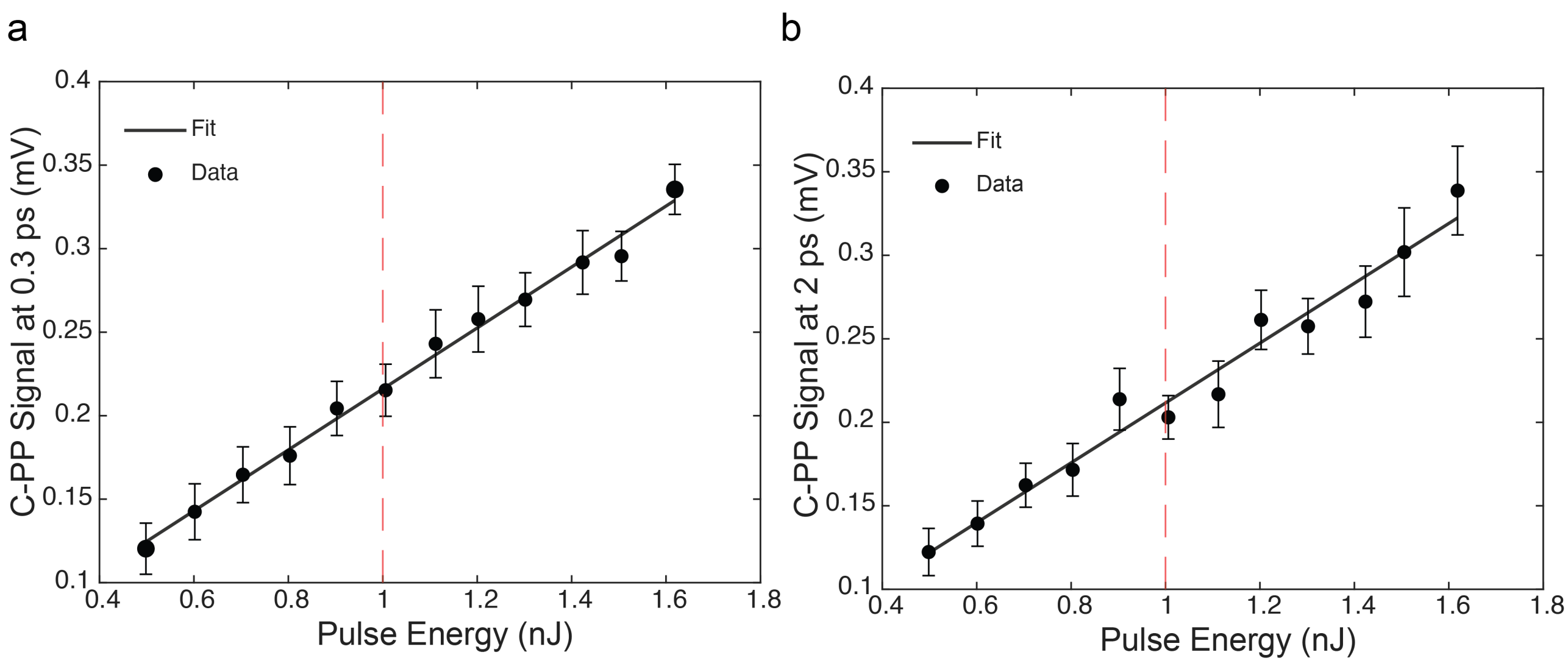

Figure S6: Pulse energy dependence of the nonlinear signal. (a) (b) C-PP signal at two pump-probe delays (T = 0.3 ps and 2 ps) has been shown as a function of pump pulse energy. The probe pulse energy was fixed at 0.9 nJ. The linear fit confirms that at 1 nJ pulse energy (used in the experiment), we are well inside the linear regime to avoid higher-order signal contributions.

References

(1) Gonzalez-Migoni, S.; Jansen, T. L. C. Fluorescence-detected two-dimensional electronic spectroscopy: A coarse-grained simulation approach. *The Journal of Chemical Physics* **2026**, *164*, 104112.

(2) Jansen, T. l. C.; Knoester, J. Nonadiabatic Effects in the Two-Dimensional Infrared Spectra of Peptides: Application to Alanine Dipeptide. *The Journal of Physical Chemistry B* **2006**, *110*, 22910–22916.

(3) Zhong, K.; Nguyen, H. L.; Do, T. N.; Tan, H.-S.; Knoester, J.; Jansen, T. L. C. Coarse-Grained Approach to Simulate Signatures of Excitation Energy Transfer in Two-Dimensional Electronic Spectroscopy of Large Molecular Systems. *Journal of Chemical Theory and Computation* **2024**, *20*, 6111–6124.

(4) Domínguez-Martín, M. A.; Sauer, P. V.; Kirst, H.; Sutter, M.; Bína, D.; Greber, B. J.; Nogales, E.; Polívka, T.; Kerfeld, C. A. Structures of a Phycobilisome in Light-Harvesting and Photoprotected States. *Nature* **2022**, *609*, 835–845.

(5) Mukamel, S.; Abramavicius, D. Many-Body Approaches for Simulating Coherent Nonlinear Spectroscopies of Electronic and Vibrational Excitons. *Chemical Reviews* **2004**, *104*, 2073–2098.

(6) Espinoza Cangahuala, M. K.; Krishnaswamy, S. R.; Kuevda, A. V.; Pshenichnikov, M. S.; Jansen, T. L. C. The first step of cyanine dye self-assembly: Dimerization. *The Journal of Chemical Physics* **2025**, *162*, 054311.

(7) Haverkort, F.; Stradomska, A.; Knoester, J. First-Principles Simulations of the Initial Phase of Self-Aggregation of a Cyanine Dye: Structure and Optical Spectra. *The Journal of Physical Chemistry B* **2014**, *118*, 8877–8890, PMID: 24979675.

(8) Kunsel, T.; Tiwari, V.; Matutes, Y. A.; Gardiner, A. T.; Cogdell, R. J.; Ogilvie, J. P.; Jansen, T. L. Simulating Fluorescence-Detected Two-Dimensional Electronic Spectroscopy of Multichromophoric Systems. *Journal of Physical Chemistry B* **2019**, *123*, 394–406.

(9) May, V.; Kühn, O. *Charge and Energy Transfer Dynamics in Molecular Systems*, 2nd ed.; Wiley-VCH: Weinheim, 2008.

(10) Womick, J. M.; Moran, A. M. Vibronic enhancement of exciton sizes and energy transport in photosynthetic complexes. *Journal of Physical Chemistry B* **2011**, *115*, 1347–1356.

(11) Stadnichuk, I. N.; Krasilnikov, P. M. Relationship between Non-Photochemical Quenching Efficiency and the Energy Transfer Rate from Phycobilisomes to Photosystem II. *Photosynthesis Research* **2024**, *159*, 177–189.

(12) Hochstrasser, R. M. Two-Dimensional IR-Spectroscopy: Polarization Anisotropy Effects. *Chemical Physics* **2001**, *266*, 273–284.

(13) Doukas, A. G.; Stefancic, V.; Buchert, J.; Alfano, R.; Zilinskas, B. A. EXCITON ANNIHILATION IN THE ISOLATED PHYCOBILIPROTEINS FROM THE BLUE-GREEN ALGA NOSTOC SP. USING PICOSECOND ABSORPTION SPECTROSCOPY. *Photochemistry and Photobiology* **1981**, *34*, 505–510.

(14) Wong, D.; Pellegrino, F.; Alfano, R. R.; Zilinskas, B. A. FLUORESCENCE RELAXATION KINETICS AND QUANTUM YIELD FROM THE ISOLATED PHYCOBILIPROTEINS OF THE BLUE-GREEN ALGA NOSTOC SP. MEASURED AS A FUNCTION OF SINGLE PICOSECOND PULSE INTENSITY, I. *Photochemistry and Photobiology* **1981**, *33*, 651–662.

(15) Grabowski, J.; Gantt, E. PHOTOPHYSICAL PROPERTIES OF PHYCOBILIPROTEINS FROM PHYCOBILISOMES: FLUORESCENCE LIFETIMES, QUANTUM

YIELDS, AND POLARIZATION SPECTRA. *Photochemistry and Photobiology* **1978**, *28*, 39–45.

(16) Tekavec, P. F.; Lott, G. A.; Marcus, A. H. Fluorescence-detected two-dimensional electronic coherence spectroscopy by acousto-optic phase modulation. *The Journal of Chemical Physics* **2007**, *127*, 214307.

(17) Sahu, A.; Bhat, V. N.; Patra, S.; Tiwari, V. High-sensitivity fluorescence-detected multidimensional electronic spectroscopy through continuous pump–probe delay scan. *The Journal of Chemical Physics* **2023**, *158*, 024201.

(18) Bhat, V. N.; Thomas, A. S.; Bhattacharyya, A.; Tiwari, V. Rapid scan white light pump–probe spectroscopy with 100 kHz shot-to-shot detection. *Optics Continuum* **2023**, *2*, 1981–1995.

(19) Thomas, A. S.; Bhat, V. N.; Tiwari, V. Rapid scan white light two-dimensional electronic spectroscopy with 100 kHz shot-to-shot detection. *The Journal of Chemical Physics* **2023**, *159*, 244202.

(20) Malý, P.; Lüttig, J.; Rose, P. A.; Turkin, A.; Lambert, C.; Krich, J. J.; Brixner, T. Separating single- from multi-particle dynamics in nonlinear spectroscopy. *Nature* **2023**, *616*, 280–287.

(21) Charvátová, K.; Malý, P. Spectro-temporal symmetry in action-detected optical spectroscopy: Highlighting excited-state dynamics in large systems. *The Journal of Chemical Physics* **2025**, *162*, 124204.

(22) Snellenburg, J. J.; Laptenok, S.; Seger, R.; Mullen, K. M.; van Stokkum, I. H. M. Glotaran: A Java-Based Graphical User Interface for the R Package TIMP. *Journal of Statistical Software* **2012**, *49*, 1–22.

(23) Stokkum, I. H. V.; Larsen, D. S.; Grondelle, R. V. Global and target analysis of time-resolved spectra. *Biochimica et Biophysica Acta (BBA) - Bioenergetics* **2004**, *1657*, 82–104.

(24) Yeh, S. W.; Ong, L. J.; Glazer, A. N.; Clark, J. H. Fluorescence properties of allophycocyanin and a crosslinked allophycocyanin trimer. *Cytometry* **1987**, *8*, 91–95.

(25) Homoelle, B. J.; Edington, M. D.; Diffey, W. M.; Beck, W. F. Stimulated Photon-Echo and Transient-Grating Studies of Protein-Matrix Solvation Dynamics and Interexciton-State Radiationless Decay in α Phycocyanin and Allophycocyanin. *The Journal of Physical Chemistry B* **1998**, *102*, 3044–3052.

(26) Womick, J. M.; Moran, A. M. Exciton coherence and energy transport in the light-harvesting dimers of allophycocyanin. *Journal of Physical Chemistry B* **2009**, *113*, 15747–15759.
